# Synthesis of Planar Chiral Ferroceneformaldehydes via Rh(I)-Catalyzed C–H Arylation

**DOI:** 10.1021/acscentsci.3c01225

**Published:** 2023-10-18

**Authors:** Hailey Hendricks, Jose. B. Roque

**Affiliations:** Department of Chemistry, Princeton University, Princeton, New Jersey, 08544, United States

In this issue of *ACS Central Science*, You and co-workers describe the development of an enantioselective rhodium(I)-catalyzed C–H arylation of ferrocene formaldehydes, enabling a versatile synthetic entry point to access 1,2-disubstituted chiral planar ferrocene derivatives.^1^ Planar chiral ferrocenes represent privileged ligands and catalysts for asymmetric catalysis. Beyond catalysis, global research efforts have been devoted to ferroquine, a ferrocene-containing drug, and related derivatives as promising candidates for antimalarial agents.^3^ Given their widespread applications, methods to access planar chiral ferrocene derivatives are in high demand.

**Figure 1:**
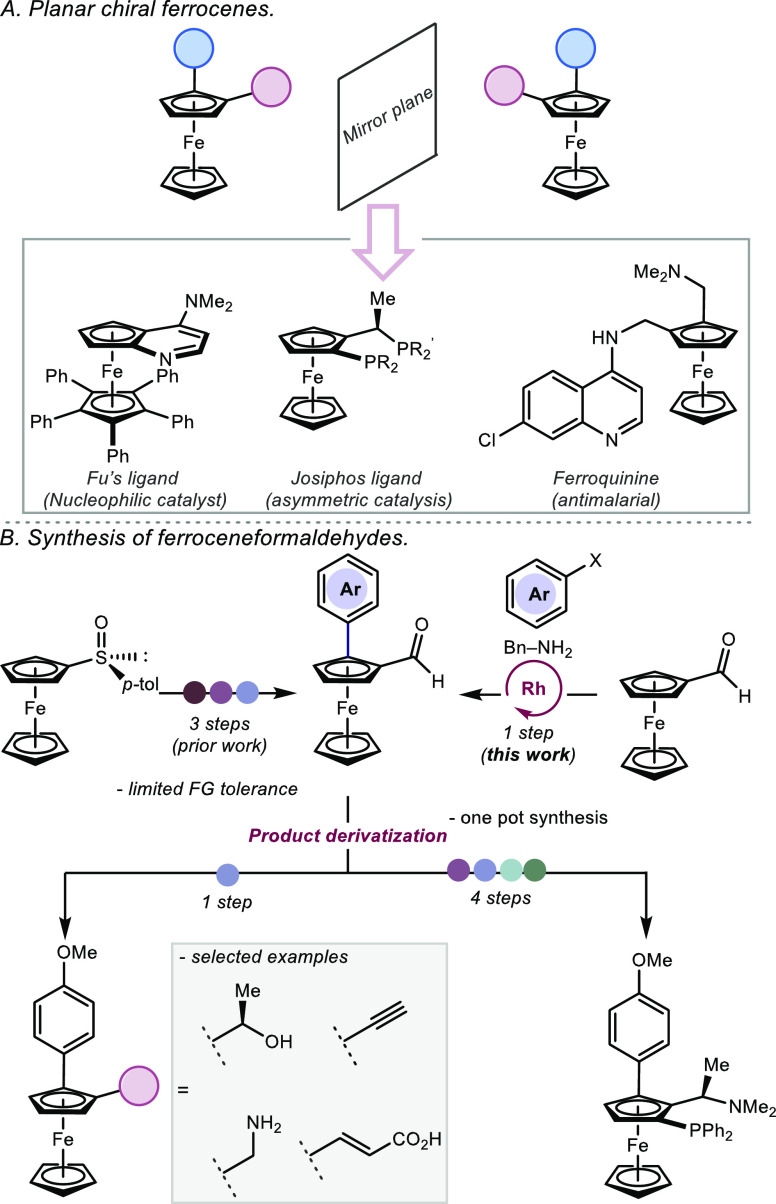
A) Planar chiral ferrocenes. B) Synthesis of ferroceneformaldehydes.

Historically, the synthesis of 1,2-disubstituted planar chiral ferrocenes has relied on the covalent attachment of a stochiometric chiral auxiliary followed by diastereoselective functionalization. For example, Ugi’s amine can mediate diastereoselective *ortho*-lithiation, which, upon trapping with a suitable electrophile, furnishes planar chiral ferrocene derivatives.^4^ Despite providing access to a wide range of phosphorus-containing ligands, the strongly basic conditions can lead to functional group incompatibility. Mild transition-metal-catalyzed C–H activation methods have expanded synthetic disconnection strategies by enabling the direct conversion of ubiquitous carbon–hydrogen bonds ito carbon–carbon and carbon–heteroatom bonds. Although several directed C–H functionalization strategies have been disclosed for accessing 1,2-disubstituted ferrocene carbonyl derivatives, they remain challenging targets due to the requirement of preinstalled chiral auxiliaries or strongly coordinating directing groups and require difficult-to-remove protecting groups, ultimately limiting product diversification.^5^ Recently, Jin and co-workers reported a palladium-catalyzed enantioselective C(sp^2^)–H arylation of ferrocenyl ketones using inexpensive l-*tert*-leucine as a chiral transient directing group.^6^ Planar chiral ferroceneformaldehydes represent an ideal starting material, given that the aldehyde group can be converted to a plethora of different functional groups using well-established methodologies. However, employing aldehydes as directing groups represents a significant challenge in rhodium-catalyzed C–H activation reactions given their weak coordinating ability and propensity to undergo competitive aldehydic C–H bond activation.^7^ If a successful enantioselective C–H functionalization of ferrocenyl aldehydes were to be realized, it would allow general access to 1,2-disubstituted chiral planar ferrocenes upon product diversification. You and co-workers report a one-pot protocol for the C–H arylation of ferrocenyl aldehydes catalyzed by a chiral phosphoramidite supported Rh(I) catalyst with a diverse set of aryl halides.^1^ The group demonstrated the synthetic versatility of the aldehyde functional group by accessing a wide range of derivatives without a loss of enantioselectivity in one step and reporting an efficient protocol to access chiral (aminoferrocenyl)phosphine ligands (Figure 1B).

**Figure 2:**
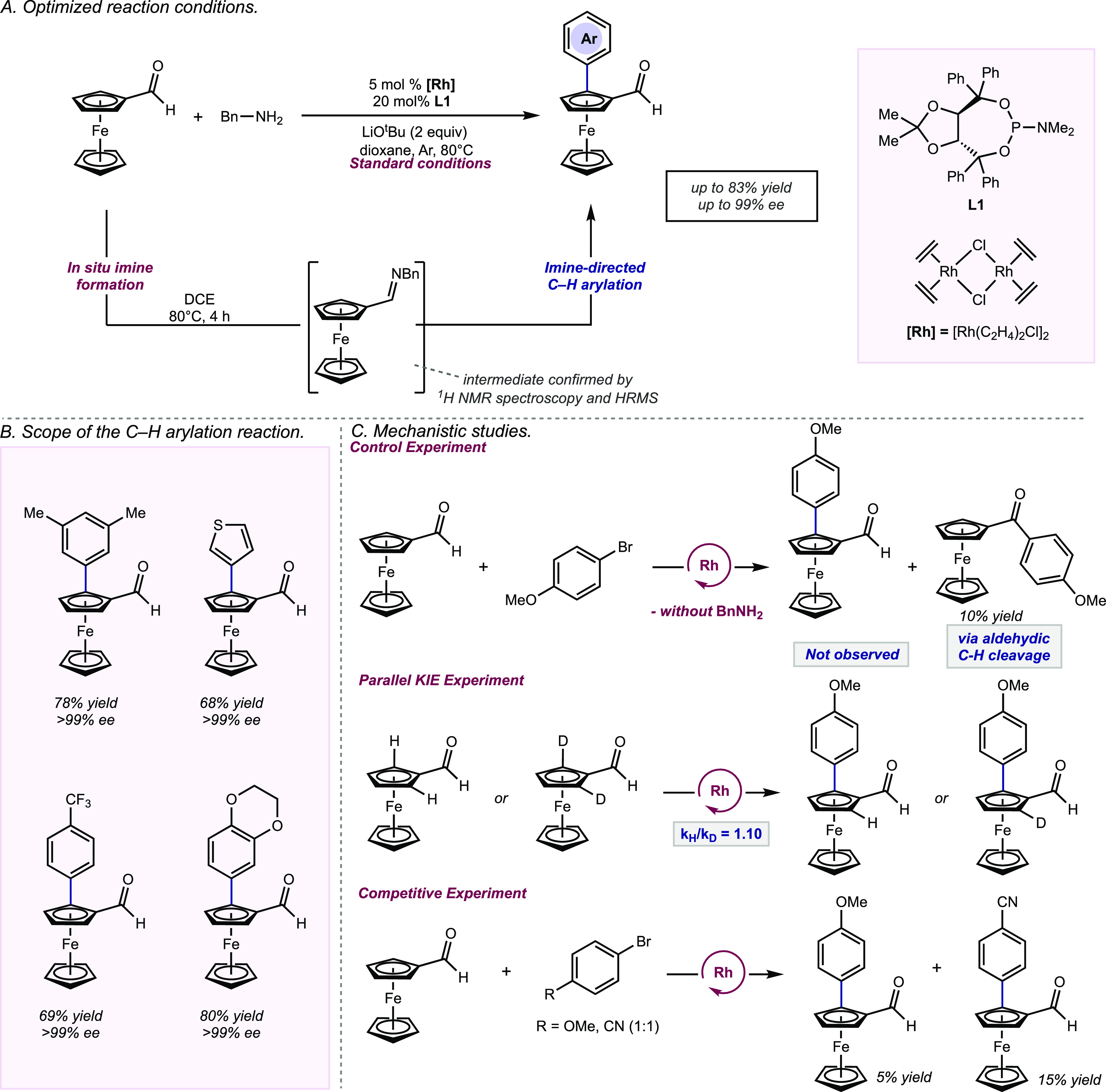
A) Optimized reaction conditions. B) Scope of the C–H arylation reaction. C) Mechanistic studies.

After extensive reaction optimization, standard conditions for catalytic experiments involved *in situ* generation of the imine by treatment of 1 equivalent of ferroceneformaldehyde with 1.1 equivalents of benzyl amine in dichloroethane at 80 °C for 4 hours. Upon removal of solvent and the addition of 2 equivalents of aryl halide with 5 mol % of rhodium(I) precursor (**[Rh]**) and 20 mol % of phosphoramidite ligand (**L1**) in dioxane at 80 °C, the desired product is furnished in high yield and high enantioselectivity (up to 83% yield and >99% ee, Figure 2A). A wide range of aryl bromide coupling partners with substituents of varying electronic influence at the *meta* and *para* positions were tolerated (Figure 2B). Functional groups such as esters, ketones, thioethers, silanes, and heteroaromatics did not adversely affect the yield of the C–H arylation. Aryl chlorides and aryl iodides could also be used as coupling partners; however, lower levels of enantioselectivity were observed with aryl iodides. To demonstrate the synthetic utility of the method, a gram-scale reaction was conducted with 2 mol % [Rh] affording the desired product in 70% yield and >99% ee.

A catalytic cycle was proposed based on a series of mechanistic experiments (Figure 2C). Control experiments support the formation of an imine intermediate as a key step in the reaction. Under standard conditions without the inclusion of a benzyl amine, the desired product was not observed, and products derived from aldehydic C–H bond cleavage were observed. Monitoring the reaction by ^1^H NMR spectroscopy and HRMS confirmed the generation of the imine intermediate. The measurement of kinetic isotope effects (KIEs) can be a powerful mechanistic tool for unearthing insights into chemical transformations involving the cleavage of C–H bonds. A parallel KIE experiment was conducted to assess if C–H cleavage is rate-determining, and the KIE was determined to be 1.10 (*k*_H_/*k*_D_), indicating that C–H cleavage is not the rate-determining step. Intermolecular and intramolecular competition kinetic isotope experiments can provide insight into the reversibility of the C–H cleavage step within the catalytic cycle. Competition experiments were conducted between 4-bromoanisole and 4-bromobenzonitrile, and the electron-poor substrate (4-bromobenzonitrile) reacted preferentially. Additional mechanistic experiments are required to determine the rate-determining step. In line with prior mechanistic studies from the group on Rh(I)-catalyzed C–H arylation using the 2-pyridinyl directing group, the authors proposed a catalytic cycle consisting of dehydration to form imine intermediate, imine-directed C–H activation via concerted metalation–deprotonation followed by oxidative addition and reductive elimination to give 1,2-disubstituted ferrocenyl imine which upon hydrolysis generates the desired product.^8^

In conclusion, the development of a Rh(I)-catalyzed enantioselective C–H arylation provided a general entry into chiral 1,2-disubstituted ferrocenes. Drawing on the laboratory’s extensive experience and enduring interest in ferrocene-containing molecules, the current study represents an exciting development with significant synthetic potential.
